# Language and face in interactions: emotion perception, social meanings, and communicative intentions

**DOI:** 10.3389/fpsyg.2023.1146494

**Published:** 2023-05-02

**Authors:** Mingya Liu, Juliane Schwab, Ursula Hess

**Affiliations:** ^1^Department of English and American Studies, Humboldt-Universität zu Berlin, Berlin, Germany; ^2^Department of Linguistics, Universität Tübingen, Tübingen, Germany; ^3^Department of Psychology, Humboldt-Universität zu Berlin, Berlin, Germany

**Keywords:** emotion, emotive marker, facial expression, interlocutor relation, social meaning, communicative intention

## Abstract

**Introduction:**

Human emotions can be complex to interpret as they have multiple sources and are often times ambiguous, for example, when the signals sent by different channels of communication are inconsistent. Our study investigates the interaction of linguistic and facial expressions of emotions.

**Methods:**

In two experiments, participants read short scenarios in German containing a direct utterance with positive or negative emotive markers, in combination with different facial expressions as still images of the speaker (i.e., the protagonist in the story). They answered questions about their perception regarding the intensity of the emotions (e.g., happiness, sadness), the properties of the expresser (e.g., honesty, warmth, likeability) and their relation to the addressee (e.g., closeness), as well as the expresser intention (e.g., irony, joke).

**Results:**

The findings suggest that facial expressions have a more dominant role in the emotion perception in comparison to emotive markers. Furthermore, consistent and inconsistent combinations of emotive markers and facial expressions convey distinct social meanings and communicative intentions.

**Conclusion:**

This research points to the importance to consider emotive markers in the emotional context that they occur in.

## 1. Introduction

Humans are social animals with the ability to share their own emotions and attend to those of others. In language-based communication, it is readily apparent that interlocutors’ exchanges often center not only around sharing information about how the world is (*It is raining*) but also around sharing one’s emotions about that fact (*Ah damn, it is raining*). Expressing emotions can help us to, for example, cope with challenging situations and to establish social relations. Recognizing others’ emotions can help us to plan what we say and what we do (Niedenthal and Brauer, 2012). However, human emotions are complex and can be difficult to recognize or identify as their expressions are multimodal and their meanings often times ambiguous – for example, certain emotion expressions are underspecified and thus context-dependent, or the signals sent by different channels of communication are inconsistent (Hess and Hareli, 2014). This paper addresses the interaction of emotion expressions in the verbal and nonverbal domains.

Linguistically speaking, there is a diverse set of expressions functioning as emotive markers, which differ in grammatical and functional properties. By definition, emotive markers are “a morpheme, syntax, or prosody that encodes the speaker’s emotive attitude toward some proposition made salient by the utterance in which it occurs, and does so in backgrounded, not-at-issue content” (Rett, 2021, p. 307). The most obvious subset of emotive markers across languages are evaluative adjectives or adverbs (Liu, 2012), as illustrated in (1) and (2). However, even constructions that do not contain explicit emotive markers can express speaker emotions, such as the so-called counterfactual optatives in (3), where the speaker expresses the emotion of regret that Mike did not pass the driving test, see a linguistic analysis of such constructions in Grosz (2012). Furthermore, a more recently established feature of human communication is the use of face emojis as in (4), which clearly express emotions; their meaning contributions, in particular, in interaction with the linguistic context, are currently under investigation (Grosz, 2022).

(1) *It’s great that Mike passed the driving test.*
(2) *Fortunately, Mike passed the driving test.*
(3) *If only Mike had passed the driving test!*
(4) *Mike passed the driving test* 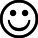
!

Emotions can also be expressed nonverbally. Nonverbal communication is generally defined as the aspect of communication that is not expressed in words (Hess, 2015). As such, it often accompanies spoken language in the form of gestures and facial expressions (Burgoon et al., 2011) that can support the meaning of the spoken word (e.g., when one shows a disgusted expression while speaking about the forgotten leftovers found recently in the fridge), or contradict the spoken word (e.g., when one uses a word of effusive praise with a malicious smile). Whereas it is well established that emotive markers play a role in conveying human emotions, much less is known about the reverse process – how do emotion expressions in nonverbal domains impact the understanding of linguistic statements?

Finally, human communication is multimodal, that is, verbal and nonverbal channels of emotion expression may be combined. At first glance, (5) describes a positive event and (6) a negative event; this is what all the existing semantic theories of evaluative adverbs would predict, as the choice of the words, in this case, the positive or negative adverb, commits the speaker to the expressed evaluation or emotion. In the theoretical linguistic literature (Potts, 2005; Simons et al., 2010; Liu, 2012), sentences such as (5) and (6) have been analyzed to express a main (i.e., at-issue) proposition P and a secondary (i.e., non-at-issue) evaluative content, non-at-issue as the content of the adverb does not address the question under discussion (QUD), in contrast to that of the matrix clause (Roberts, 1996/2012). Accordingly, both (5) and (6) are felicitous answers to the QUD of whether Mike passed the driving test. However, if the QUD is, e.g., whether it is fortunate whether Mike passed the driving test, (5) would be an infelicitous answer in comparison to “It is fortunate that he did.” We will get back to this distinction later in the paper. Crucially, linguistic theories are usually not concerned with the question of whether the expressed evaluation is genuine or not, with exceptions of work on figurative speeches including irony (a.o., Sperber and Wilson, 1981; Noveck, 2018, Chapter 11).

(5) *Fortunately, Mike passed the driving test.*
a. At-issue content: Mike passed the driving test. (P)
b. Non-at-issue content: fortunate(P)
(6) *Unfortunately, Mike did not pass the driving test.*
a. At-issue content: Mike did not the driving test. (not-P)
b. Non-at-issue content: unfortunate(not-P)

However, if the speaker’s facial expression does not match the emotive marker, a different impression can be gained. Failing to show a positive expression in (5) may denote disinterest in Mike and his test. Showing a happy expression in (6) suggests *schadenfreude*, the malicious pleasure in seeing Mike fail, maybe because he bragged about his prowess at the wheel before the test. These examples show that the facial emotions accompanying a sentence can moderate the perception of that sentence (Krumhuber and Manstead, 2009). A positive statement accompanied by a sad face appears less clearly positive and may even turn negative, and vice versa. Yet, matters are even more complicated and interesting than this. The smile is the most ubiquitous emotion expression but it is also complex (Hess et al., 2002). Although smiles generally are perceived as signaling affiliative intent (Knutson, 1996), this is not always the case. In addition to schadenfreude, smiles may denote prideful superiority or submission (Niedenthal et al., 2010). But they can also signal pity or empathic concern. These smiles can often be distinguished by their appearance (Hess et al., 2002; Martin et al., 2017). Thus, semantic emotions (as encoded in language) and visual emotions *via* faces can be consistent or inconsistent, and different combinations may generate different inferences regarding emotion perception, interlocutor relationship, social meanings, and communicative intentions.

Lastly, while it is plausible that the facial expression of the speaker can modify the interpretation of a given statement, it is also important to note that faces do not only play a role in spoken language (signed languages included) but also for language in the written format. Think of the still images accompanying newspaper articles, e.g., images of athletes after a major win or lose, or images of medal winners – the sheer joy displayed in the “chosen” still image of the gold medalist in comparison to images of the silver and bronze medalists, or an image of the ones who did not win a medal, is an effective frame for the news article. Or think of the still images of politicians belonging to different parties that newspapers choose to use – presenting the respective politician in more or less favorable light depending on, among others, the newspaper’s own political affinity and the evaluation of newsworthiness. Perhaps most obvious in its intention to affect readers’ perception (and subsequent purchasing decision) is the use of faces in (print) advertising (Xiao and Ding, 2014).

In this paper, we investigate sentences with emotive markers as carriers of semantic (linguistic) emotions, in interaction with different facial expressions (visual emotions). We aim to address (1) the distributional constraints of these two modalities (i.e., to what extent do the directions of linguistic statements and of facial expressions need to match?), (2) the impact of facial expressions on the interpretation and perception of linguistic statements, as well as (3) the overall pragmatic (i.e., social meaning, e.g., speaker genuineness and speaker-addressee closeness) effects of different combinations. This work builds on the idea that people interpret both facial expressions and linguistic choices (of, e.g., a linguistic variety, or the use of an optional expression as in the case of this paper) as indices of the expresser’s personal traits and relations to the audience (Lambert et al., 1960; Hareli and Hess, 2010; Burnett, 2019; Kastendieck et al., 2021). In addressing the modulating role of facial expressions in the perception of linguistic statements, our findings contribute to the understanding of multimodal human communication – we focus on the combination of still images and linguistic utterances in the written format in this paper as the first step before looking at the combination of dynamic faces and linguistic utterances in the oral format as the next step. Moreover, we consider individual differences in comprehenders’ (i.e., study participants’) social and communicative skills [measured with a subset of scales from the German Autism Spectrum Quotient (AQ); Baron-Cohen et al., 2001] and ability to recognize and describe emotions [measured by the German Toronto Alexithymia Scale 26 (TAS-26; Kupfer et al., 2000)]. This allows us to address how variation in individuals’ processing of emotive markers and facial expressions influences their perception and interpretation of multimodal communicative signals.

The following section reports two experiments in German on sentences with emotive markers in interaction with facial expressions, conducted to investigate the distributional constraints of their co-occurrence and how different combinations affect the perception of the emotion, the speaker and the utterance. In both experiments, participants read short scenarios containing contextual information, followed by a direct utterance without or with a facial expression as still image of the speaker (i.e., the protagonist in the story). They then answer questions about their perception by keypress on a 7-point Likert scale, concerning the intensity of the emotions (e.g., happiness, sadness), the properties of the expresser *per se* (e.g., honesty, warmth, likability) and their relation to the addressee (e.g., closeness), as well as their communicative intentions, i.e., whether they convey literal or nonliteral meanings (e.g., irony, joke). As the results of these experiments closely relate to each other, we delay the critical discussion of our findings until after Experiment 2.

## 2. Experiments

Our study was based on the following hypotheses:

*H1*: Emotive markers and facial expressions modulate the emotion perception.

*H2*: Facial expressions can reduce or add ambiguity in the emotion perception of linguistic statements, and convey different social meanings (about the expresser and interlocutor relation) and inferences about the communicative intentions of the expresser.

More specifically, our predictions were:

1. The combination of the positive emotive marker and a happy face would increase the happiness ratings, and vice versa for the negative marker and the sad face.
2. Consistent combinations of the emotive marker and the facial expression would lead to a more positive perception about the expresser and their relation to the addressee than inconsistent combinations.
3. Inconsistent combinations of the emotive marker and the facial expression would lead to a higher proportion of non-literal interpretations of the communicative intentions than consistent combinations.

The two experiments we report below were both implemented online^1^ with the PennController for Internet Based Experiments (Zehr and Schwarz, 2018). Participants were recruited through the online crowd-sourcing platform Prolific^2^ and received monetary compensation for their participation. We received informed consent from all participants.

### 2.1. Experiment 1

#### 2.1.1. Participants

We initially recruited 130 participants, 13 of which were removed from the final data analysis for failing attention checks (less than 7 out of 9 *yes/no* comprehension questions on filler items were answered correctly). All remaining 117 participants (51 male, 1 non-binary, 65 female, aged 18–65) were German native speakers.

#### 2.1.2. Materials

We used 18 items (scenarios), each comprising 3 sentences, see (7) for an example. The first two sentences were context-setting and remained the same across all experimental conditions. The third sentence consisted of a speaker’s utterance, which was always accompanied by a still image of the speaker’s face. The experimental manipulation consisted of systematic manipulations of this sentence in a 3 × 3 design with the factors ‘face’ (i.e., facial expression) and ‘marker’ (i.e., emotive marker): The still image of the speaker showed either a ‘happy’, ‘sad’ or ‘neutral’ facial expression. Images were taken from the Amsterdam Dynamic Facial Expression Set, which has been validated for emotion recognition rates (van der Schalk et al., 2011). Furthermore, the speaker’s utterance either stood alone or contained a sentence-initial marker indicating positive (*super* ‘great’, *zum Glück* ‘luckily’) or negative (*schade* ‘too bad’, *leider* ‘unfortunately’) emotions.

(7)
S1: Dennis hat drei Kinder. (Dennis has three children.)
S2: Er sagt zu seiner Frau: (He says to his wife:)
S3: “{Super, heute /Schade, heute /Heute} ist der letzte Schultag.”
(“Great, today/Too bad, today/Today is the last school day.”)

Additionally, we included 9 filler items. Fillers did not include an emotive marker in the third sentence. Three of the fillers were accompanied by a happy facial expression, three by a sad facial expression, and three by a neutral one.

We used a Latin square design to ensure that participants only saw each item once (in one of its experimental conditions). The ratio of male and female speakers was balanced across experimental items and fillers. We used 5 female and 5 male models from the Amsterdam Dynamic Facial Expression Set. The pairing between these images and the experimental items was randomized across participants so that the results are not confounded by inherent properties of the faces.

#### 2.1.3. Procedure

**Figure 1:**
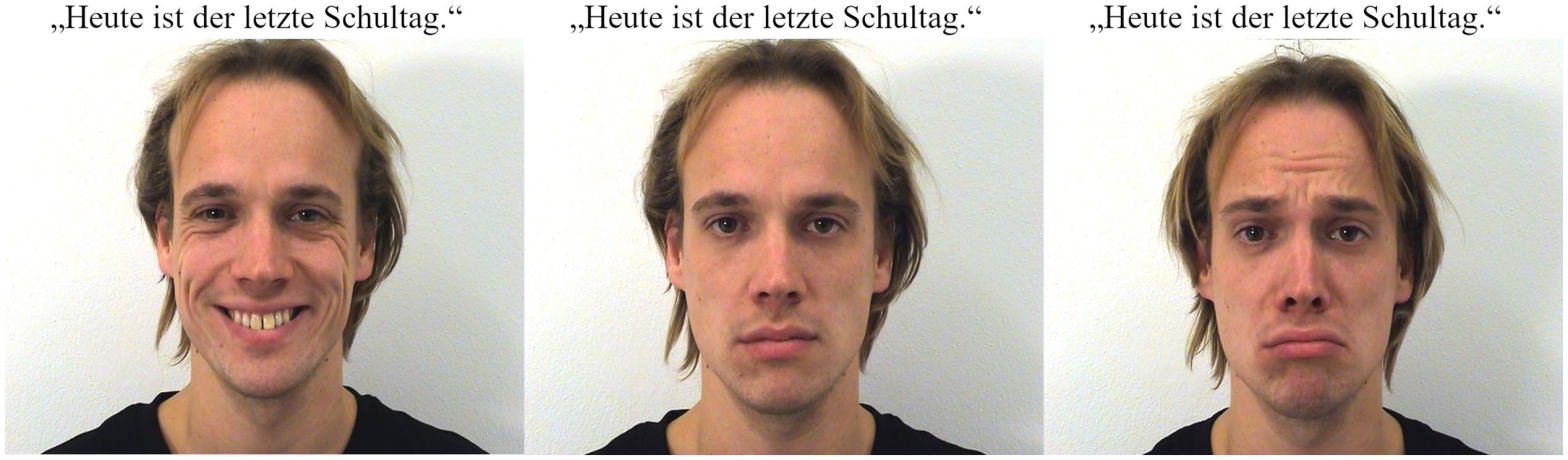
Example of the joint presentation of a target sentence and speaker image (showing happy, neutral, and sad facial expressions, respectively) during experimental trials. Image source: Amsterdam Dynamic Facial Expression Set (van der Schalk et al., 2011). Reproduced with permission.

On each trial (fillers and experimental trials), participants first had to read the three sentences as in (7). The sentences would appear one by one on separate screens. To finish reading a sentence and proceed to the next one, participants had to press the space bar. Only the final sentence was accompanied by a still image of the speaker’s face, appearing at the same time as the sentence itself (see Figure 1).

Upon pressing the space bar after the final sentence, participants were shown three questions, one after the other on separate screens: In the first question (of emotion perception), they were asked to rate how strongly they thought the speaker [e.g., Dennis in (7)] felt happy, sad, or neutral with respect to their utterance.^3^ Participants could provide ratings on each of these emotions using 7-point Likert scales from 1 = *not at all* to 7 = *very strongly.* The order in which these three affective states were presented was counterbalanced across participants. In the second question (of speaker perception), participants were asked to rate how honest they felt the speaker [e.g., Dennis in (7)] was about their feelings toward the content of the utterance. Again, they provided ratings on a 7-point Likert scales from 1 = *not at all honest* to 7 = *very honest.* Finally, in the third question (of interlocutor relation), participants were asked to rate how closely they felt speaker and listener [which involved various interlocutor relations in different situational contexts, e.g., Dennis and his wife in (7)] were, again providing ratings on a 7-point Likert scale from 1 = *not at all close* to 7 = *very close*.

In contrast to the experimental items, filler items only included the first and the second questions (emotion and honesty ratings), with the third question being replaced by a *yes/no* comprehension question about the contents of the item participants had just read.

#### 2.1.4. Data analysis

Data were analyzed in R, version 4.0 (R Development Core Team, 2020) using mixed effects cumulative link models for ordinal regression (package *ordinal*). All models used by-subject and by-item random intercepts. We did not use random slopes as these models would not converge. The factors ‘face’ and ‘marker’ were entered as treatment coded factors with their levels ‘neutral face’ and ‘no marker’ as reference. The interaction between these two factors was determined using the Likelihood Ratio Test (package *drop1*).

#### 2.1.5. Results

##### 2.1.5.1. Question 1: emotion perception

**Figure 2:**
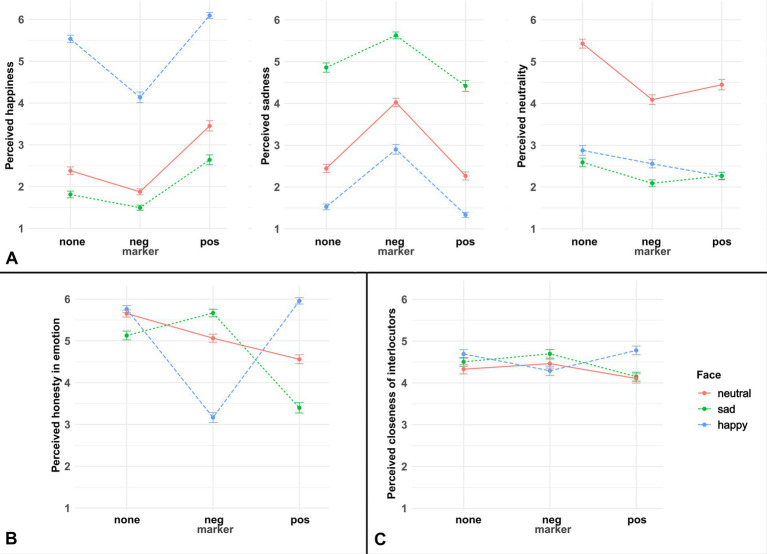
Mean ratings for all questions in Experiment 1, with error bars showing standard errors around the mean. Panel **(A)** of this figure shows responses on the emotion perception questions, panel **(B)** on the speaker perception question, and panel **(C)** on the interlocutor relation question.

The results are plotted in Figure 2A.

###### 2.1.5.1.1. Happiness

The Likelihood Ratio Test indicated a significant interaction between face and marker (LRT = 21.89, df = 4, *p* = 0.0002),^4^ with the effect of facial expressions on happiness ratings being bigger than the effect of emotive markers. Compared to the reference condition (no marker + neutral face), conditions with a positive marker (*ß* = 1.14, SE = 0.17, *p* < 0.0001) or happy face (*ß* = 3.51, SE = 0.18, *p* < 0.0001) elicited higher happiness ratings, whereas conditions with a negative marker (*ß* = −0.68, SE = 0.17, *p* < 0.0001) or sad face (*ß* = −0.90, SE = 0.17, *p* < 0.0001) elicited lower happiness ratings. Additionally, there was a significant interaction between the happy face and the negative marker, as the latter decreased happiness ratings more strongly if the speaker showed a happy face than if they showed a neutral face (*ß* = −0.95, SE = 0.24, *p* < 0.0001). This may in part be due to the fact that the neutral face condition is already perceived as expressing low happiness, regardless of the presence of an additional negative marker.

###### 2.1.5.1.2. Sadness

The Likelihood Ratio Test indicated a significant interaction between face and marker (LRT = 31.44, df = 4, *p* < 0.0001), with the effect of facial expressions on sadness ratings being bigger than the effect of emotive markers. Compared to the reference condition (no marker + neutral face), conditions with a negative marker (*ß* = 1.76, SE = 0.17, *p* < 0.0001) or sad face (*ß* = 2.77, SE = 0.18, *p* < 0.0001) elicited higher sadness ratings. By contrast, although a happy face decreased sadness ratings (*ß* = −1.59, SE = 0.17, *p* < 0.0001), the positive marker had no significant effect (*p* = 0.13). Additionally, there was a significant interaction between the sad face and the negative marker, as the latter increased sadness ratings more strongly if the speaker showed a neutral facial expression than if they showed a sad facial expression (*ß* = −0.85, SE = 0.24, *p* = 0.0003). This suggests a greater role of the facial compared to the linguistic expression of emotion.

###### 2.1.5.1.3. Neutrality

The Likelihood Ratio Test indicated a significant interaction between face and marker (LRT = 39.09, df = 4, *p* < 0.0001), with the effect of facial expressions on neutrality ratings being bigger than the effect of emotive markers. Compared to the reference condition (no marker + neutral face), conditions with either marker (positive: *ß* = −1.32, SE = 0.18, *p* < 0.0001, negative: *ß* = −1.70, SE = 0.18, *p* < 0.0001) or facial expression (happy: *ß* = −3.14, SE = 0.19, *p* < 0.0001, sad: *ß* = −3.47, SE = 0.18, *p* < 0.0001) elicited lower neutrality ratings. Additionally, there were significant interactions between both happy and sad faces and positive and negative markers, such that the effect of markers was weaker if the speaker showed a happy/sad facial expression than if the speaker showed a neutral face (all *p*-values < 0.05).

##### 2.1.5.2. Question 2: speaker perception (honesty)

The results are plotted in Figure 2B. The Likelihood Ratio Test indicated a significant interaction between face and marker (LRT = 598.64, df = 4, *p* < 0.0001), with idiosyncratic effects for matching and mismatching combinations of facial expressions and emotive markers. Specifically, compared to the reference condition (no marker + neutral face), conditions with either marker (positive: *ß* = −1.37, SE = 0.17, *p* < 0.0001, negative: *ß* = −0.80, SE = 0.17, *p* < 0.0001) elicited lower honesty ratings. Moreover, the condition with a negative face was rated as less honest than the reference condition (*ß* = −0.63, SE = 0.17, *p* < 0.0001, whereas the happy face had no significant effect on honesty ratings (*p* = 0.20). Additionally, there were significant interactions between both happy and sad faces and positive and negative markers (all *p*-values < 0.01). This is due to two factors: firstly, the presence of matching positive or negative markers with happy or sad faces increased honesty ratings, whereas they decreased honesty ratings if paired with a neutral face (see above). Secondly, the presence of mismatching negative or positive markers with happy or sad faces decreased honesty ratings more strongly than if they were paired with a neutral facial expression.

##### 2.1.5.3. Question 3: interlocutor relation (closeness)

The results are plotted in Figure 2C. The Likelihood Ratio Test indicated a significant interaction between face and marker (LRT = 43.04, df = 4, *p* < 0.0001), with prominent differences between neutral and sad facial expressions compared to the happy facial expression. Specifically, compared to the reference condition (no marker + neutral face), conditions with a happy face elicited higher closeness ratings (*ß* = 0.63, SE = 0.17, *p* = 0.0003), while there were no significant effects of sad faces or either type of marker. Additionally, there was a significant interaction between the positive face and the negative marker (*ß* = −0.72, SE = 0.24, *p* = 0.003), such the addition of the latter decreased closeness ratings if the speaker showed a (mismatching) happy facial expression, whereas it had no effect on closeness ratings if the speaker showed a neutral facial expression.

### 2.2. Experiment 2

#### 2.2.1. Participants

We initially recruited 120 participants, 3 of which were removed from the final data analysis for failing attention checks (less than 7 out of 9 *yes/no* comprehension questions on filler items were answered correctly). All remaining 117 participants (67 male, 50 female, aged 18–59) were German native speakers.

#### 2.2.2. Materials

As in the previous experiment, we used 18 items, each comprising 3 sentences. 15 of those items were the same as in Experiment 1, see (7); the other 3 items were replaced from the previous study as we assumed that their contexts may have an emotional bias (e.g., one took place at the speaker’s birthday party [positive emotional bias], the second established that the speaker was being treated at a hospital [negative emotional bias], the third took place as conversation in anticipation of attending a concert [positive emotional bias]). As before, our critical manipulation consisted of systematic changes in the third sentence: In a 3 × 3 design with the factors ‘face’ and ‘marker’, the sentence now appeared either without an image of the speaker’s face, or with a still image of the speaker showing either a ‘happy’ or ‘sad’ face. Furthermore, the speaker’s utterance either stood alone, or contained a sentence-initial emotive marker indicating (*super* ‘great’, *zum Glück* ‘luckily’) or negative (*schade* ‘too bad’, *leider* ‘unfortunately’) emotions. Additionally, we included the same 9 filler items as in Experiment 1.

The ratio of male and female speakers was balanced across experimental items and fillers. We used 5 female and 5 male models from the Amsterdam Dynamic Facial Expression Set, the same ones as in Experiment 1. The pairing between these images and the experimental items was randomized across participants. Crucially, the experiment was structured in two blocks to avoid confusion over the fact that some trials appear with an image of the speaker while others do not. Blocks were designed so that one block (comprising 6 experimental items and 3 fillers) contained all trials on which the third sentence was not accompanied by a still image of the speaker’s face, whereas the other block (comprising the remaining 12 experimental items and 6 fillers) contained all trials in which it was. The order of these blocks was counterbalanced across participants.

Experiment 2 differed from Experiment 1 in three aspects: First, neutral faces were not used in Experiment 2, as the results of Experiment 1 show that they might not be sufficiently “neutral” after all to function as ideal baselines, see Figure 1, where neutral faces patterned more like sad faces in the happiness ratings. We expected the results to replicate those in Experiment 1. Secondly, we adjusted the rating questions by dropping the “neutrality” rating, and enriched the experiment by measures of speaker characteristics (from honesty to warmth, likability, etc.) and a multiple-choice task for the speaker’s communicative intentions. With the latter two additions, we aimed to identify the involved social meanings in a more fine-grained manner and the additional pragmatic inferences more directly. Finally, for explorative purposes, we included two additional measures of individual differences: One was part of the German Autism Spectrum Quotient (AQ; Baron-Cohen et al., 2001), specifically the subscales measuring individuals’ social and communicative skills. Individual differences on these measures have previously been associated with differences in the processing of pragmatic language phenomena such as scalar implicatures (Nieuwland et al., 2010) and irony (Spotorno and Noveck, 2014). Participants who scored higher on these AQ subscales were shown to have more difficulty with the relevant pragmatic phenomena. The other measure of individual differences targeted participants’ ability to recognize and describe emotions (in others and themselves), for which we used the German Toronto Alexithymia Scale 26 (TAS-26; Kupfer et al., 2000).

#### 2.2.3. Procedure

On each trial (fillers and experimental trials), participants first had to read the three sentences as those in (7). The sentences would appear one by one on separate screens. To finish reading a sentence and proceed to the next one, participants had to press the space bar. Only the final sentence was accompanied by a still image of the speaker’s face (on the relevant experimental conditions), appearing at the same time as the sentence itself.

Upon pressing the space bar after the final sentence, participants were shown three questions, one after the other on separate screens: In the first question (of emotion perception), they were asked to rate how strongly they thought the speaker felt happiness or sadness with respect to their utterance. Participants could provide ratings on both of these emotions using 7-point Likert scales from 1 = *not at all* to 7 = *very strongly*. The order in which these two emotions were presented was counterbalanced across participants.

In the second question (of speaker perception), participants were asked to evaluate properties of the speaker on four semantic differentials (using 7-point scales): *kalt* (‘cold’) – *warm* (‘warm’), *unsymphatisch* (‘unlikable’) – *sympathisch* (‘likable’), *unangemessen* (‘inappropriate’) – *angemessen* (‘appropriate’), and *unehrlich* (‘dishonest’) – *ehrlich* (‘honest’), adjusted from the measures used in, e.g., Kastendieck et al. (2021). The order in which these differentials were presented was counterbalanced across participants.

Finally, in the third question (of communicative intentions), participants were asked to indicate what they thought the speaker was intending to express with their utterance. They were asked to tick all options that they thought applied from a list of 7 options: *ehrliche Freude* (‘sincere happiness’), *ehrliche Traurigkeit* (‘sincere sadness’), *Witz/Scherz* (‘joke/jest’), *Ironie/Sarkasmus* (‘irony/sarcasm’), *Schadenfreude* (‘schadenfreude’), *Zuversicht/Stolz* (‘confidence/pride’), and *Ärger/Irritation* (‘anger/irritation’). The first two options were predicted to be the most salient interpretation for matching combinations of facial expressions and emotive markers, whereas *Witz*/Scherz (‘joke/jest’), *Ironie/Sarkasmus* (‘irony/sarcasm’), and *Schadenfreude* (‘schadenfreude’) were predicted to be more salient for mismatching combinations. The last two options were included as filler responses, which were not predicted to be salient interpretations on any condition. The order in which these options were shown was counterbalanced across participants.

In contrast to the experimental items, filler items additionally included a *yes/no* comprehension question about the contents of the item participants had just read. This was included between the ratings of the speaker’s happiness/sadness and the ratings of the speaker’s properties.

The two AQ subscales and TAS-26 were included at the end of the experiment, as two questionnaires on separate screens. The order of the two questionnaires was counterbalanced across participants.

#### 2.2.4. Data analysis

Data were analyzed in R, version 4.0. All scalar responses (happiness ratings, sadness ratings, semantic differentials) were analyzed using mixed effects cumulative link models for ordinal regression (package *ordinal*). The binary responses on the labeling task (option ticked/not ticked) were analyzed using mixed effects binary logistic regression models (package *lme4*). All models used by-subject and by-item random intercepts. We did not include random slopes as these models would not converge. The factors ‘face’ and ‘marker’ were entered as treatment coded factors with their levels ‘no face’ and ‘no marker’ as reference. In addition, we included individual participants’ scores on the (combined) AQ subscales and TAS-26 as numerical fixed effects along with their interaction with the two other factors. Interaction effects were determined using the Likelihood Ratio Test (package *drop1*). Pairwise comparisons between conditions of interest were conducted using the *emmeans* package, with all extracted *p*-values being Tukey-adjusted for multiple comparisons.

#### 2.2.5. Results

##### 2.2.5.1. Question 1: emotion perception

**Figure 3:**
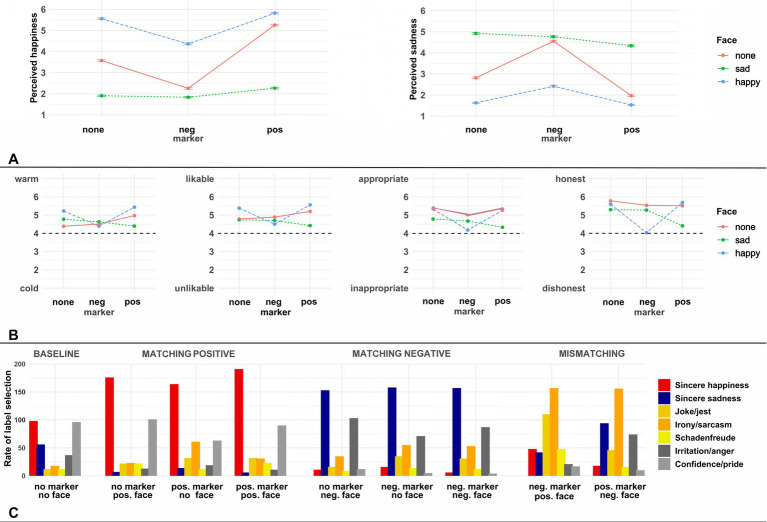
Results for all questions in Experiment 2. Panel **(A)** of this figure shows the mean scale ratings on the emotion perception questions, with error bars showing standard errors around the mean. Panel **(B)** displays mean ratings on the four semantic differentials addressing speaker perception, again with error bars showing standard errors around the mean. Panel **(C)** shows the rate (in total numbers) at which each of the seven potential utterance labels were selected for each condition in the question addressing communicative intentions.

The results are plotted in Figure 3A.

###### 2.2.5.1.1. Happiness

Compared to the reference condition (no marker + neutral face), conditions with a negative marker (*ß* = −1.64, SE = 0.28, *p* < 0.0001) or sad face (*ß* = −2.63, SE = 0.29, *p* < 0.0001) elicited lower happiness ratings, whereas conditions with a positive marker (*ß* = 2.18, SE = 0.29, *p* < 0.0001) or happy face (*ß* = 1.07, SE = 0.29, *p* < 0.0001) elicited higher happiness ratings. The AQ subscales (*p* = 0.07) and TAS-26 (*p* = 0.39) scores had no significant marginal effects. Significant interactions between the sad face and positive/negative markers (all *p*-values < 0.001) indicate that the addition of an emotive marker had a stronger effect on increasing (respectively decreasing) happiness ratings in the conditions where no still image of the speaker’s face was present compared to the conditions in which a sad face was displayed. By contrast, the happy face only interacted with positive markers (*ß* = −1.12, SE = 0.41, *p* = 0.007), such that positive markers increased happiness ratings more strongly in the absence of a still image of the speaker’s face compared to the conditions with a happy face. There was no interaction with negative markers (*p* = 0.99). TAS-26 scores additionally significantly interacted with happy faces (*ß* = 0.03, SE = 0.01, *p* = 0.0002), such that individuals with higher TAS-26 scores were more likely to provide high happiness ratings for conditions with a happy face compared to the conditions without any still image of the speaker’s face. The Likelihood Ratio Test indicated no three-way interaction involving TAS-26 scores; but it did indicate an interaction involving AQ subscale scores, face and marker (LRT = 29.26, df = 4, *p* < 0.0001). Teasing this apart, the model suggested two lower-level two-way interactions involving AQ subscale scores, one with sad faces (*ß* = 0.04, SE = 0.02, *p* = 0.012) and one with positive markers (*ß* = −0.04, SE =0.016, *p* = 0.008): in these conditions, compared to the reference condition without marker or face, individuals with higher AQ subscale scores provided lower happiness ratings.

###### 2.2.5.1.2. Sadness

Mirroring the happiness ratings, the reference condition elicited lower sadness ratings compared to conditions with a negative marker (*ß* = 1.58, SE = 0.28, *p* < 0.0001) or sad face (ß = 2.09, SE = 0.28, *p* < 0.0001), whereas it elicited higher sadness ratings than conditions with a positive marker (*ß* = −1.72, SE = 0.31, *p* < 0.0001) or happy face (*ß* = −0.98, SE = 0.32, *p* = 0.003). The AQ subscales (*p* = 0.59) and TAS-26 (*p* = 0.18) scores had no significant marginal effects. Significant interactions between the sad face and positive/negative markers (all *p*-values < 0.01) indicate that the addition of an emotive marker had a stronger effect on increasing (respectively decreasing) sadness ratings in the conditions where no still image of the speaker’s face was present compared to the conditions in which a sad face was displayed. By contrast, the happy face only interacted with positive markers (*ß* = 1.20, SE = 0.48, *p* = 0.012) – decreasing sadness ratings more strongly for conditions without still image of the speaker –, while the presence of a negative marker did not interact with the happy face (*p* = 0.99). TAS-26 scores additionally significantly interacted with sad faces (ß = 0.02, SE = 0.01, *p* = 0.007), such that individuals with higher TAS-26 scores were more likely to provide high sadness ratings for conditions with a sad face compared to the conditions without any still image of the speaker’s face. AQ subscale scores interacted with both happy (*ß* = −0.04, SE = 0.02, *p* = 0.012) and sad (*ß* = −0.07, SE = 0.02, *p* < 0.0001) faces, suggesting that individuals with higher AQ subscale scores were less strongly affected by facial expressions in their sadness ratings. The Likelihood Ratio Test indicated no further three-way interaction involving TAS-26 or AQ subscale scores (all *p*-values > 0.05).

The rating results of the emotion perception in both experiments are summarized in Table 1. By and large, Experiment 1 replicated the findings of Experiment 1, providing convergent evidence for the effects of face and marker and the dominant role of face. Experiment 1 and 2 partly differed on the interaction effects, which may be attributable to differences in the respective baselines (Exp.1: neutral face, Exp.2: no speaker face).

**Table 1:** Comparison of results on the emotion perception questions in Experiments 1 and 2.

Effect	Experiment 1		Experiment 2	
	Happiness rating	Sadness rating	Happiness rating	Sadness rating
Face effects	↑ Happy face ↓ Sad face	↑ Sad face ↓ Happy face	↑ Happy face ↓ Sad face	↑ Sad face ↓ Happy face
Marker effects	↑ Positive marker ↓ Negative marker	↑ Negative marker ↓ Positive marker	↑ Positive marker ↓ Negative marker	↑ Negative marker ↓ Positive marker
Interactions	*Happy face × negative marker* (marker effect stronger than with neutral face)	*Sad face × negative marker* (marker effect weaker than with neutral face)	*Sad face × positive marker* *Sad face × negative marker* *Happy face × positive marker* (marker effects weaker than without speaker face)	*Sad face × positive marker* *Sad face × negative marker* *Happy face × positive marker* (marker effects weaker than without speaker face)
	–	–	*AQ × sad face* (bigger effect of sad face) *AQ × positive marker* (smaller effect of positive marker)	*TAS-26 × sad face* (bigger effect of sad face) *AQ × happy face* *AQ × sad face* (smaller effects of happy/sad face)

##### 2.2.5.2. Question 2: speaker perception

The results are plotted in Figure 3B. A principal component analysis (PCA), using the R function *prcomp*, with the four semantic differentials indicated a single component which explained 75.6% of the variance when grouping the responses on the differentials from *kalt* (‘cold’) – *warm* (‘warm’) and *unsymphatisch* (‘unlikable’) – *sympathisch* (‘likable’), whereas there was a single component that explained 72.7% of the variance when grouping the responses on the differentials *unangemessen* (‘inappropriate’) – *angemessen* (‘appropriate’), and *unehrlich* (‘dishonest’) – *ehrlich* (‘honest’). Grouping the responses even further, into a single composite response, however, would likely miss important underlying variance, as the primary component explained only 58% of the variance. For analysis, we therefore combined the responses to these questions into two composite variables labelled ‘warmth/likability’ and ‘honesty/appropriateness’. An analysis in which we analyzed the four responses separately can be found in the Supplementary materials.

###### 2.2.5.2.1. Warmth/likability

Compared to the reference condition (no marker + neutral face), conditions with a negative marker (*ß* = 0.21, SE = 0.06, *p* = 0.0009) or sad face (*ß* = 0.33, SE = 0.06, *p* < 0.0001) elicited lower ratings for speaker *warmth/likability*, whereas conditions with a positive marker (*ß* = 0.89, SE = 0.06, *p* < 0.0001) or happy face (*ß* = 1.41, SE = 0.06, *p* < 0.0001) elicited higher ratings. There were no significant marginal effects of TAS-26 or AQ subscale scores. Interactions between both faces (happy/sad) and markers (positive/negative) (all *p*-values < 0.001) indicate, on the one hand, that the addition of a positive or negative marker (compared to no marker) in conditions without speaker image increased *warmth/likability* ratings but had little effect on the ratings in conditions with a matching (happy/sad) face. Moreover, the addition of a positive or negative marker reduced *warmth/likability* ratings for sentences accompanied by a mismatching sad (respectively happy) face. The Likelihood Ratio Test additionally indicated two three-way interactions between face, marker, and TAS-26 scores (LRT = 26.78, df = 4, *p* < 0.0001), and between face, marker and AQ subscale scores (LRT = 16.29, df = 4, *p* = 0.003). Specifically, higher AQ subscale scores were associated with bigger effect sizes on the interaction between sad faces and (positive/negative) markers, suggesting that they raised (respectively lowered) their *warmth/likability* ratings more strongly for matching/mismatching combinations of sad faces and markers in comparison to conditions in which only either face or marker were present. The reverse held true for higher TAS-26 scores, suggesting they treated the sad face conditions more like the baseline conditions without speaker image.

###### 2.2.5.2.2. Honesty/appropriateness

Compared to the reference condition (no marker + neutral face), conditions with a negative marker (*ß* = −0.57, SE = 0.06, *p* < 0.0001), positive marker (*ß* = −0.26, SE = 0.06, *p* < 0.0001) or sad face (*ß* = −0.94, SE = 0.06, *p* < 0.0001) all elicited lower *honesty/appropriateness* ratings. There were no significant marginal effects of happy faces, TAS-26 or AQ subscale scores. Interactions between both facial expressions (happy/sad) and both markers (positive/negative) (all *p*-values < 0.01) indicate that the addition of a positive or negative marker decreased *honesty/appropriateness* ratings if the sentence was associated with a mismatching still image of the speaker’s face (compared to conditions without speaker image), whereas the addition of markers matching the face had no effect on the ratings. The Likelihood Ratio Test additionally indicated a three-way interaction between face, marker, and AQ subscale scores (LRT = 37.38, df = 4, *p* < 0.0001), Specifically, higher AQ subscale scores were associated with bigger effect sizes on the interaction between positive markers and happy/sad faces, meaning that their *honesty/appropriateness* ratings for conditions with sad/happy faces differed more strongly from the ratings provided to the baseline condition without speaker image in as far as the effect of adding a positive marker is concerned.

##### 2.2.5.3. Question 3: communicative intentions

The results are plotted in Figure 3C. The selection rates for communicative intentions across the nine experimental conditions were grouped into three categories for analysis: *sincere happiness, sincere sadness,* and *non-literal* (including *joke/jest, irony/sarcasm, schadenfreude*). Analyses of the filler labels (*confidence/pride*, *irritation/anger*) are not reported here, but an additional analysis that separately targeted each of the seven utterance labels can be found in the Supplementary materials.

Compared to the reference condition, participants were significantly less likely to label a speaker’s utterance as expressing *sincere happiness* if it was associated with a negative marker (*ß* = 2.63, SE = 0.33, *p* < 0.0001) or sad face (*ß* = 2.98, SE = 0.36, *p* < 0.0001), whereas the pattern was reversed for positive markers (*ß* = −1.37, SE = 0.21, *p* < 0.0001) and happy faces (*ß* = −1.71, SE = 0.22, *p* < 0.0001). In addition, interactions between happy faces and positive markers (*ß* = 0.96, SE = 0.32, *p* = 0.003), as well as sad faces and negative markers (*ß* = −2.02, SE = 0.63, *p* = 0.001), indicate that the addition of an emotive marker exerted most of its effect in the conditions without still image of the speaker’s face, whereas their addition to sentences associated with matching sad or happy faces did not substantially alter the proportion to which participants labeled the utterance as expressing *sincere happiness*.

A similar pattern emerges for *sincere sadness*, for which negative markers (*ß* = −2.34, SE = 0.24, *p* < 0.0001) or sad faces (*ß* = −2.30, SE = 0.24, *p* < 0.0001) increased the proportion to which this label was chosen, whereas positive markers (*ß* = 1.76, SE = 0.34, *p* < 0.0001) or happy faces (*ß* = 2.88, SE = 0.52, *p* < 0.0001) decreased it. Interactions between matching happy faces and positive markers (*ß* = −1.60, SE = 0.80, *p* = 0.045), as well as sad faces and negative markers (*ß* = 2.24, SE = 0.32, *p* < 0.0001), mirror the previous finding, in that the addition of a matching marker had little effect on the proportion to which conditions with happy or sad faces were labeled as expressing *sincere sadness*. An interaction between happy faces and TAS-26 scores (*ß* = −0.92, SE = 0.44, *p* = 0.039) further suggested that the effect of the happy face was smaller for individuals with higher TAS-26 scores.

For the combined non-literal labels, conditions with either a negative marker (*ß* = −1.08, SE = 0.20, *p* < 0.0001), positive marker (*ß* = −1.06, SE = 0.20, p < 0.0001) or happy face (*ß* = −0.54, SE = 0.21, *p* = 0.012) were all more likely to be labeled as intending a nonliteral interpretation than the reference condition. There was no marginal effect of sad faces – possibly because our nonliteral options were of a more jovial nature, associating them with positive expressions more than with negative facial expressions. Interactions between both adjectives (positive/negative) and faces (happy/sad) (all *p*-values < 0.05) indicated that, on the one hand, mismatching combinations of faces and markers were substantially more likely to be labeled as expressing a nonliteral interpretation than conditions in which only one of these cues was present, while, on the other hand, matching combinations of faces and markers did not substantially alter the rate of nonliteral interpretations compared to conditions in which only a single cue is present.

## 3. General discussion

Communicative meanings result from an interplay of verbal and nonverbal information. Although there is a large body of literature on all of these communicative tools, the relation between them remains far from clear. In this paper, we reported two experiments tackling the interaction between linguistic and facial expressions as carriers of human emotions. Both experiments used emotion ratings and ratings of social meanings (i.e., ratings about speaker properties); in addition, the second experiment extended the investigation to the question of communicative intentions and individual differences. Our experiments confirmed the hypotheses H1 and H2 about the effects of emotive markers and facial expressions in emotion perception. In particular, they indicated a modulating function of emotional facial expressions in the perception of linguistic statements and pragmatic inferences about speaker properties and their communicative intentions depending on the relation between face and language. In the following we will summarize the main findings and discuss them in detail.

The findings of Experiment 1 were: (1) While both emotive markers and facial expressions were shown to affect emotion perception significantly, the effect of facial expressions was stronger, consistently across all the three ratings of happiness, sadness and neutrality. (2) The results of the honesty and closeness ratings showed that consistent combinations of facial expressions and emotive markers increased the impression of speaker honesty and interlocutor-closeness, in opposition to inconsistent ones. The findings of Experiment 2 were: (1) The results of the emotion perception questions were similar to those in Experiment 1. The results of the happiness and the sadness ratings very much were the opposite of each other, without the interference of the neutral faces, *cf.* Figure 2 vs. Figure 3; Table 1. (2) Using an enriched set of speaker properties, we found a similar result with regard to social meanings as in Experiment 1, in that consistent combinations led to a more positive speaker perception compared to inconsistent combinations. (3) Furthermore, consistent combinations of face and language were found to associate with more literal interpretations of communicative intentions than inconsistent ones.

First, we discuss the larger effect of faces than emotive markers. Wierzbicka (1996, 1999), for example, argues that the meaning of facial expressions can be analyzed similarly to the meaning of words. While the semantics of faces is generally more restricted than that of words, we focused on linguistic and visual expressions of emotion in our study, a meaning dimension where face and language are more comparable. The assumption about the face-language parallelism would predict an effect of both cues but not the dominant role of faces in emotion perception. We believe that there are several non-exclusive explanations for this. First, facial expressions are more primitive and unconscious in comparison to linguistic expressions, that is, choice of words involves more controlled and conscious processes. Therefore, we might be able to suppress our emotions by not saying something or saying something more indirectly whereas faking facial emotions (e.g., showing a sad face while being happy, or vice versa) is more difficult and meanwhile easier to detect. Along these lines, our results could mean that facial expressions are a more honest window to the inner emotional states of the expresser, and accordingly, comprehenders rely more on these in emotion perception than on words. This finding provides convergent evidence that in human communication, facial expressions are a key source of information about the emotions of others (Hess et al., 1988; Fridlund and Russell, 2006). Obscured emotion expressions, e.g., by a speaker wearing a surgical mask, tend to be recognized less well than in uncovered faces among adults (Carbon, 2020; Grundmann et al., 2021; see Calbi et al., 2021, for an exception), and even if they are recognized well, the expressions are perceived as less intense (Kastendieck et al., 2021). This, along with our results on the respective roles of facial and linguistic expressions in emotion perception helps us to understand better the challenges of a changed communicative context due to the wearing of medical masks during the global COVID-19 pandemic.

While we hold the above explanation about the primacy of facial expressions of emotion as plausible, we cannot rule out other explanations. In particular, one alternative possibility lies in the respective salience of linguistic and visual information. As we mentioned in the introduction, evaluative adverbs such as *leider* have been analyzed as triggers of non-at-issue meanings. That is, their occurrence in a sentence presupposes that the evaluative content does not target the main QUD, making it a secondary – and thereby backgrounded – meaning. In the nonverbal domain, some researchers have also applied such analyses to speech-accompanying iconic gestures in interactive situations. Ebert (2014), for example, relates gestures like that of indicating the size (“this tall”) of the referred object while saying “Have you seen my coffee mug?” to triggers of non-at-issue meaning. In this way, the conveyed overall meaning of speech and gesture in the example boils down to similarly saying “Have you seen my coffee mug, which is this tall?.” For the moment, it is unclear to us whether and what facial expressions, in which contexts of occurrence, could be approached similarly – putting into question whether linguistic expressions of emotion (e.g., in the form of evaluative adverbs) are thus inherently more backgrounded than facial expressions. Furthermore, the still images used in our study were obviously salient due to their size and appearance, see Figure 1. The contrast between the visually foregrounded status of the face and the linguistically backgrounded status of the emotive marker might therefore have contributed to the dominant effect of facial expressions in emotion perception observed in our study.

A further related possibility is that the emotive markers we used in our study are not necessarily emotive or speaker-oriented. In the linguistic literature, evaluative adverbs are sometimes called speaker-oriented adverbs (e.g., Jackendoff, 1972; Ernst, 2009). However, they do not always express the speaker’s emotions (see Liu, 2012). In the minimal pair in (8), the evaluation in (8a) associates with the speaker’s emotion, whereas in (8b) it is an evaluation of the state of affairs, which the speaker judges as sad for Sandra or some other people. The speaker might be empathetic, but they might as well not be, as the sentence can be continued as “…but I am happy, as I am a fan of Chardonnay.” In other words, whether evaluative adverbs reveal the speaker’s inner state or whether it is a device of politeness (e.g., showing public empathy) depends on the perspective taking in their context of use. This ambiguity might have contributed to the perceived intensity of emotions. In this regard, it is worth mentioning that the German *leider* ‘unfortunately’ might be more inherently more emotive or more speaker-oriented than *zum Glück*, as it is fine to anchor toward a perspectival agent other than the speaker with the latter but odd with the former (Zum Glück/*Leider für euch bin ich heute da. ‘Fortunately/Unfortunately for you, I am here today.’) For this reason, we took a look at the data of the four emotive markers we used in the study; the descriptive statistics, however, suggests no clear picture. Thus, we will leave this for proper investigation in future.

(8)
a. *Sadly (for me/us), it did not work out in the end.*
b. *Sadly for Sandra/some, Chardonnay beats Riesling as a summer drink.*

In addition to the possible interpretations of the data regarding the emotion perception, our results are not only useful in understanding the multimodal feature of human communication and the function of facial expressions therein, but they have additional implications for linguistics. As we mentioned in Section 1, the existing semantic theories of evaluative adverbs or emotive markers focus on their literal interpretation (i.e., compositional semantics and pragmatics). Our findings show that in addition to irony with intonational support or support of linguistic context, the modulating role of faces needs to be considered in the investigation of non-literal vs. literal meanings (Hancock, 2004). Both experiments show that alignments of face and language give rise to higher ratings of speaker appropriateness, honesty, etc., and more straightforward interpretations of their communicative intentions, whereas mismatches lead to more negative speaker perceptions and more non-literal interpretations of their communicative intentions. Language and face interact in various manners so that comprehenders look for contextually relevant interpretations even in the case of apparent inconsistency or inappropriateness (see Attardo, 2000 for a theory of irony based on the idea that an ironical utterance is both inappropriate and relevant to its context). The mechanism behind this can arguably be captured *via* Gricean reasoning, as we do with linguistic pragmatic phenomena such as irony, which has been argued to arise *via* a violation of the maxim of quality, i.e., saying something that is literally false (see Noveck, 2018 for a recent concise review). We will not spell out the exact mechanism by which “apparently inconsistent” face-speech combinations give rise to contextually relevant pragmatic inferences for listeners. But we speculate that such a mechanism might involve the violation of the maxim of manner (i.e., avoid obscurity or ambiguity). In this respect, a related question is whether all inconsistent combinations allow for pragmatic reanalysis. Our answer is negative, as we have the intuition that sentences such as “Es ist schade, dass heute der letzte Schultag ist” (It is too bad that today is the last day of the school) with a happy face are more difficult to repair in comparison to the versions used in our study, “Schade, heute ist der letzte Schultag” (Too bad that today is the last day of the school). Further studying face-speech combinations on these closely related utterance forms will not only shed light on the role of faces but also on the mechanisms of meaning composition in different linguistic structures.

While the results above are not surprising with the named explanations calling for future investigation, some results turned out to be unexpected. The first is that of neutral faces in Experiment 1. The results showed that they pattern more toward sad faces, that is, they are not really “neutral.” This raises the question what it means to have a neutral face. In social interactions, it is possible that a more “friendly” face than the “neutral” face we used is the social norm and a deviation from that would receive negative interpretations. On the other hand, what counts as a neutral or normal face is dependent on multiple factors relating to inter-cultural, inter-individual and intra-individual variation.

Before we conclude the paper, we would like to briefly address the scope and limitations of the current study. In natural communication, we see dynamic faces whose expressions can change from second to second. In our study, participants saw still face images staying on the screen with a sentence, that is, the face was not only prominent in its nature as a picture, as we discussed above, but also allowed longer processing times of the same impression. Future studies need to take this into consideration, for example, by presenting still images and sentences using the method of rapid serial visual presentation (RSVP), or by comparing still images and video clips of synthetic faces. Furthermore, whereas still images accompanying written texts are pervasive, understanding the general role of faces requires us to consider using synthetic faces instead for simulating the oral format of communication. With these factors taken into consideration, our study used the strictly controlled contexts where faces and words along the emotive dimension are combined. The finding of differences between consistent and inconsistent combinations provides novel and important perspectives on the processing of verbal and nonverbal information. Last but not the least, while Experiment 2 partially replicated the findings of Experiment 1, it also takes into account individual differences in emotion perception. Even though these components were added exploratively, they reveal that perception of language and face is probabilistic in nature and can vary with differences in social and communicative skills of comprehenders. Thus, effective communication would require interlocutors to be aware of these aspects; otherwise, miscommunication and misunderstandings can easily arise.

## 4. Conclusion

The present study is among the very few that target the pragmatic interpretations of face and language combined. It supplements the literature on non-literal meanings (e.g., due to intonation in the case of irony) and brings us forward in the understanding of multimodal communication. It provides evidence for the modulating function of facial expressions on the perception of linguistic statements. We focused on basic emotion expressions in the language and face domains. Our findings are that faces play a more dominant role than emotive markers in emotion perception. Mismatches between face and language give rise to negative social meanings in comparison to matched face and language. Given the apparent inconsistency, comprehenders look for context-relevant interpretations, which gives rise to a higher proportion of non-literal interpretations of speaker intentions.

## Data availability statement

The datasets presented in this study can be found in online repositories. The names of the repository/repositories and accession number(s) can be found at: https://osf.io/tk74w/?view_only=c4cbbff4ea9641c384bf774a0ed97d9e.

## Ethics statement

The studies involving human participants were reviewed and approved by the Ethics Committee of the German Linguistic Society (Deutsche Gesellschaft für Sprachwissenschaft, DGfS). The patients/participants provided their written informed consent to participate in this study.

## Author contributions

ML and UH conceived the current study. JS conducted the study, the data analyses, and prepared the report of both experiments. ML prepared the first draft of the manuscript with revisions from all authors. All authors designed research together and were responsible for the interpretation of the data. All authors contributed to the article and approved the submitted version.

## Funding

ML’s work was partially funded by the DFG-SFB 1412, 416591334.

## Conflict of interest

The authors declare that the research was conducted in the absence of any commercial or financial relationships that could be construed as a potential conflict of interest.

## Publisher’s note

All claims expressed in this article are solely those of the authors and do not necessarily represent those of their affiliated organizations, or those of the publisher, the editors and the reviewers. Any product that may be evaluated in this article, or claim that may be made by its manufacturer, is not guaranteed or endorsed by the publisher.

## Footnotes

^1^ https://farm.pcibex.net/

^2^ https://www.prolific.co/

^3^We included the "neutrality" measure in Experiment 1 relying on the method of Krumhuber and Manstead (2009). However, we were aware that "neutral" might be neither an emotion nor an affective state, unlike "indifferent." Due to the unclarity, we excluded the condition of "neutral faces" and the measure of "neutral emotions" in Experiment 2.

^4^We provide the results of all regression analyses in terms of the effect estimate ß, the standard error (SE) around the mean effect estimate, and the value of p indicating statistical significance of the effect. Results of Likelihood Ratio Tests are provided by their test statistic (LRT), degrees of freedom (df) in the compared models, and the value of p indicating statistical significance.
