# Supplementary material for: Language and face in interactions: emotion perception, social meanings, and communicative intentions
Source: Front Psychol. 2023 May 2;14:1146494. doi: 10.3389/fpsyg.2023.1146494 (PMC10185906; doi:10.3389/fpsyg.2023.1146494)
Supplement: Supplementary file 1 [file Data_Sheet_1.docx]

Language and Face in Interactions:

emotion perception, social meanings, and communicative intentions

Mingya Liu, Juliane Schwab, Ursula Hess

Supplementary Material

# Supplementary data analyses

## Experiment 2, question 2: speaker perception analyzed separately for each semantic differential

*Warmth.* Compared to the reference condition (no marker + neutral face), conditions with a negative marker (ß = 0.18, SE = 0.06, p < 0.0001) or sad face (ß = 0.59, SE = 0.06, p < 0.0001) elicited lower ratings on the scale from *cold* to *warm*, whereas conditions with a positive marker (ß = 0.85, SE = 0.06, p < 0.0001) or happy facial expression (ß = 1.48, SE = 0.06, p < 0.0001) elicited higher ratings. There were no significant marginal effects of TAS-26 or AQ subscale scores. Interactions between both facial expressions (happy/sad) and both markers (positive/negative) (all p’s < 0.001) indicate, on the one hand, that the addition of a positive marker (compared to no marker) increased warmth ratings if the sentence was not associated with a still image of the speaker’s face (compared to conditions with happy faces), whereas it reduced warmth ratings for sentences accompanied by a sad facial expression (a *mismatch* condition). On the other hand, the addition of a negative marker strongly decreased warmth ratings only if the sentence was accompanied by a happy face (the reverse *mismatch* condition), whereas it slightly increased warmth ratings for sentences without speaker image. The Likelihood Ratio Test additionally indicated two three-way interactions between face, marker, and TAS-26 scores (LRT = 25.64, df = 4, p < 0.0001), and between face, marker and AQ subscale scores (LRT = 13.94, df = 4, p = 0.007). Specifically, higher AQ subscale scores were associated with bigger effect sizes on the interaction between sad faces and (positive/negative) markers, whereas the reverse held true for higher TAS-26 scores; see the main paper for further discussion.

*Likability.* Compared to the reference condition (no marker + neutral face), conditions with a negative marker (ß = 0.20, SE = 0.06, p = 0.001), positive marker (ß = 0.70, SE = 0.06, p < 0.0001) or happy face (ß = 1.10, SE = 0.06, p < 0.0001) elicited higher ratings on the scale from *unlikable* to *likable*. There were no significant marginal effects of the sad face, TAS-26 or AQ subscale scores. Interactions between the happy faces and both markers (both p’s < 0.001) indicate that the addition of a positive marker (compared to no marker) increased likability ratings only if the sentence appeared without a still image of the speaker’s face, whereas the addition of a negative marker tended to decrease likability ratings only if the sentence appeared together with a (mismatching) happy face. An interaction between the sad faces and positive markers (ß = -1.09, SE = 0.09, p < 0.0001) indicated a similar effect for this mismatch, such that positive markers tended to reduce warmth ratings for sentences accompanied by a sad face. The Likelihood Ratio Test additionally indicated two three-way interactions between face, marker, and TAS-26 scores (LRT = 23.21, df = 4, df = 0.0001), and between face, marker and AQ subscale scores (LRT = 15.53, df = 4, p = 0.0037), respectively. Specifically, individuals with higher TAS-26 scores in general showed smaller effects in response to (happy/sad) faces but showed a bigger effect size on the interaction between (mismatching) happy faces and negative markers. Conversely, mirroring results on the warmth ratings, higher AQ subscale scores were associated with bigger effect sizes on the interaction between sad faces and (positive/negative) markers.

*Appropriateness.* Compared to the reference condition (no marker + neutral face), conditions with a negative marker (ß = -0.57, SE = 0.06, p < 0.0001) or sad facial expression (ß = -0.84, SE = 0.06, p < 0.0001) elicited lower ratings on the scale from *inappropriate* to *appropriate*. There were no significant marginal effects of the positive marker, happy facial expression, TAS-26 or AQ subscale scores. Interactions between both (happy/sad) facial expressions and negative markers (all p’s < 0.0001) indicated that the addition of a negative marker (compared to no marker) decreased appropriateness ratings in conditions without still image of the speaker’s face, but even more strongly so if the sentence was associated with a (mismatching) happy face. An additional interaction between sad faces and positive markers (ß = -0.54, SE = 0.09, p < 0.0001) further underscores that both mismatching combinations led to lower *appropriateness* ratings. The addition of negative markers to sentences with a (matching) sad face had no effect on appropriateness ratings and there was no interaction between happy faces and positive markers. The Likelihood Ratio Test additionally indicated a three-way interaction between face, marker, and AQ subscale scores (LRT = 14.90, df = 4, p = 0.0049), such that higher AQ subscale scores were associated with bigger effect sizes on the interaction between mismatching sad faces and positive markers. This suggests they perceived mismatching combinations of sad faces and positive markers to be more inappropriate compared to conditions with only one of these cues.

*Honesty.* Compared to the reference condition (no marker + neutral face), conditions with a negative marker (ß = -0.31, SE = 0.06, p < 0.0001), sad face (ß = -0.67, SE = 0.06, p < 0.0001), positive marker (ß = -0.34, SE = 0.06, p < 0.0001) or happy face (ß = -0.20, SE = 0.06, p = 0.002) all elicited lower ratings on the scale from *dishonest* to *honest*. There were no significant marginal effects of TAS-26 or AQ subscale scores. Interactions between both faces (happy/sad) and both markers (positive/negative) (all p’s < 0.001) indicate that the addition of a positive or negative marker decreased honesty ratings if the sentence was associated with a mismatching still image of the speaker’s face (compared to conditions without speaker image), whereas the addition of markers matching the speaker’s face had no effect on perceived honesty. The Likelihood Ratio Test additionally indicated two three-way interactions between face, marker, and AQ subscale scores (LRT = 47.96, df = 4, p < 0.0001), and between face, marker, and TAS-26 score (LRT = 16.99, df = 4, p = 0.002), respectively. Specifically, higher AQ subscale scores were associated with bigger effect sizes on the interactions between faces and markers, meaning that they more strongly raised/lowered their honesty ratings in response to matching (respectively mismatching) face and marker combinations compared to the comparison condition without either face or marker. Meanwhile, higher TAS-26 scores were associated with smaller effect sizes on the interaction between positive markers and happy faces, meaning that their honesty ratings were even less affected by the co-presence of these matching cues (compared to conditions without cues).

## Experiment 2, question 3: communicative intentions analyzed separately for each utterance label

Results for the labels *sincere happiness* and *sincere sadness* are reported in the main paper.

For *irony/sarcasm*, conditions with either a negative marker (ß = -1.39, SE = 0.30, p < 0.0001), positive marker (ß = -1.52, SE = 0.30, p < 0.0001) or sad face (ß = -0.74, SE = 0.32, p = 0.019) were all more likely to be labeled as expressing irony or sarcasm than the reference condition. There was no marginal effect of happy faces. Interactions between both adjectives (positive/negative) and facial expressions (happy/sad) (all p’s < 0.05) indicated that, on the one hand, mismatching combinations of facial expressions and markers were substantially more likely to be labeled as expressing irony/sarcasm than conditions in which only one of these cues was present, while for matching combinations of facial expressions and markers no substantial difference from the marker-only conditions was found.

For the related category of *joke/jest*, only conditions with either a positive (ß = -1.22, SE = 0.39, p = 0.001) or negative (ß = -1.33, SE = 0.38, p = 0.0005) marker significantly differed from the reference condition. An interaction between happy facial expressions and negative markers moreover indicated that the proportion to which participants labeled the utterance as expressing a joke was substantially higher when combining these mismatching cues than for each of these single cues.

For *schadenfreude*, no significant effects were found.

As expected for *irritation/anger,* conditions with either a negative marker (ß = -0.98, SE = 0.25, p < 0.0001) or sad facial expression (ß = -1.70, SE = 0.24, p < 0.0001) were more likely to be labeled as such (compared to the reference condition), whereas the pattern was reversed for positive markers (ß = 0.90, SE = 0.32, p = 0.005) or happy facial expressions (ß = 1.30, SE = 0.36, p = 0.0003). An interaction between sad facial expressions and negative markers (ß = 1.35, SE = 0.32, p < 0.0001) moreover indicated that the proportion of *irritation/anger* labels was *not* substantially altered by combining these cues.

Lastly, conditions with a positive marker (ß = 0.68, SE = 0.22, p = 0.002) were more likely to be labeled as expressing *confidence/pride*, while conditions with either a negative marker (ß = 5.58, SE = 1.48, p = 0.0002) or sad facial expressions (ß = 2.94, SE = 0.37, p < 0.0001) were less likely to be labeled as such. Interactions between negative markers and happy (ß = -3.00, SE = 1.51, p = 0.047) or sad (ß = -4.28, SE = 1.65, p = 0.0096) facial expressions indicated that the combinatory effects were driven by the marker, such that the addition of negative markers to sentences associated with a happy or sad facial expression significantly reduced the proportion of *confidence/pride* labels, albeit more so in the mismatching combination. An interaction between negative markers and AQ subscale scores (ß = 2.85, SE = 1.35, p = 0.035) suggested that individuals with higher test scores showed increased effects of the negative marker.
